# Automated Software Analysis of Fetal Movement Recorded during a Pregnant Woman’s Sleep at Home

**DOI:** 10.1371/journal.pone.0130503

**Published:** 2015-06-17

**Authors:** Kyoko Nishihara, Noboru Ohki, Hideo Kamata, Eiji Ryo, Shigeko Horiuchi

**Affiliations:** 1 Fatigue and Working Life Research Group, The Institute for Science of Labour, Kanagawa-ken, Japan; 2 NoruPro Light Systems, Tokyo, Japan; 3 Department of Obstetrics and Gynecology, Teikyo University, School of Medicine, Tokyo, Japan; 4 Department of Maternal Infant Nursing & Midwifery, St. Luke’s International University, Tokyo, Japan; 5 Integrated Brain Function Project, Tokyo Metropolitan Institute of Medical Science, Tokyo, Japan; University of Barcelona, SPAIN

## Abstract

Fetal movement is an important biological index of fetal well-being. Since 2008, we have been developing an original capacitive acceleration sensor and device that a pregnant woman can easily use to record fetal movement by herself at home during sleep. In this study, we report a newly developed automated software system for analyzing recorded fetal movement. This study will introduce the system and compare its results to those of a manual analysis of the same fetal movement signals (Experiment I). We will also demonstrate an appropriate way to use the system (Experiment II). In Experiment I, fetal movement data reported previously for six pregnant women at 28-38 gestational weeks were used. We evaluated the agreement of the manual and automated analyses for the same 10-sec epochs using prevalence-adjusted bias-adjusted kappa (PABAK) including quantitative indicators for prevalence and bias. The mean PABAK value was 0.83, which can be considered almost perfect. In Experiment II, twelve pregnant women at 24-36 gestational weeks recorded fetal movement at night once every four weeks. Overall, mean fetal movement counts per hour during maternal sleep significantly decreased along with gestational weeks, though individual differences in fetal development were noted. This newly developed automated analysis system can provide important data throughout late pregnancy.

## Introduction

Fetal movement is an important biological index of fetal well-being. Mothers can feel fetal movement starting at about 20 gestational weeks. Absence of maternal perception of fetal movement is one symptom of fetal death, and a reduction in fetal movement is an alarming sign of fetal compromise [1, 2]. In order to evaluate fetal well-being, Manning et al. proposed a fetal biophysical profile for use during ultrasound imaging [3]. That profile included fetal movement parameters, and the profile is now widely used. Detecting fetal movement and fetal heart rate with a Doppler device is now commonly known as the Non Stress Test, and its clinical contribution has been great [4, 5]. Most obstetricians and midwives check fetal movement using ultrasonography in medical facilities to confirm fetal well-being. Nevertheless, there are two stillbirths for every 1000 newborns in high-income countries, and stillbirths are a daily reality around the world [6]. According to a WHO report, there are more than 25 stillbirths per 1000 newborns in low-income countries. In addition, most stillbirths occur at home. If there were a convenient way to monitor and confirm fetal well-being at home, the stillbirth rate would decrease around the world.

Morokuma et al. have used ultrasound imaging to study fetal brain dysfunction and behavioral patterns, including decreased fetal movement parameters, in order to identify fetuses at high risk of poor neurological outcomes [7]. Using the Doppler method of evaluating fetal movement and heart rate, DiPietro et al. showed the relationship between heart rate and fetal movement on the neurological development of fetuses at low risk [8]. Both of these studies were done using a short recording time, less than one hour. Since a fetus has an ultradian rhythm [9], the findings obtained from a one-hour recording are insufficient. Long-term recordings are necessary to establish a highly accurate index of fetal development. A new system is necessary as a third option for recording fetal physiological parameters over the long term, throughout pregnancy, in order to monitor and study more about the neurological development of fetuses. Thus, long-term fetal monitoring is important not only for ensuring fetal well-being, but also for doing studies of fetal neurological development.

In 2008, we employed a newly developed capacitive acceleration sensor that could make recordings of the oscillations of the maternal abdominal wall caused by fetal movements. That study was successful in recording micro-arousals evoked by fetal movement as reflected on the mother's EEG during nocturnal sleep [10]. In addition, the study demonstrated high agreement between signals detected by the sensor and those noted by maternal perception.

In 2012, we developed a new and original recording device based on fetal movement acceleration measurement (FMAM) [11]. With this device, a pregnant woman can record fetal movement by herself at home over the long term. The FMAM device is small, lightweight, easy to use, and non-invasive. In the 2012 study, we demonstrated that gross movement recorded by the FMAM showed a high agreement with that made by ultrasonography.

Subsequently, many pregnant women were asked to record fetal movement during sleep by themselves using the FMAM recorder at home. Ryo and Kamata manually calculated the count values for fetal movement recorded by the FMAM and demonstrated that the values compared well to those calculated by ultrasonography [12]. However, there are two problems involved in counting fetal movement. One is the difficulty of excluding artifacts from the mothers’ physiological signals, such as maternal body movement, heartbeat, and breathing movement. The other problem is that manual analysis of overnight data is time-consuming.

We have now developed a new and original, automated software system for analyzing and counting fetal movements, and we are introducing it here. In Experiment I in this study, we analyzed the same fetal movement data with both manual analysis and the automated software. Because of practical difficulties and ethical issues, we could not simultaneously record with the FMAM device and ultrasonography during sleep.

In Experiment II, we analyzed newly recorded data of fetal movement week by week following gestation to establish a new fetal movement count index using the same analytical conditions as in Experiment I. Furthermore, we were able to analyze the data in more detail than with manual analysis. In Experiment II, we show that the FMAM device and its automated system of analysis are appropriate for monitoring fetal well-being.

## Methods

### 1 Using the FMAM sensor and recorder

**Fig 1:**
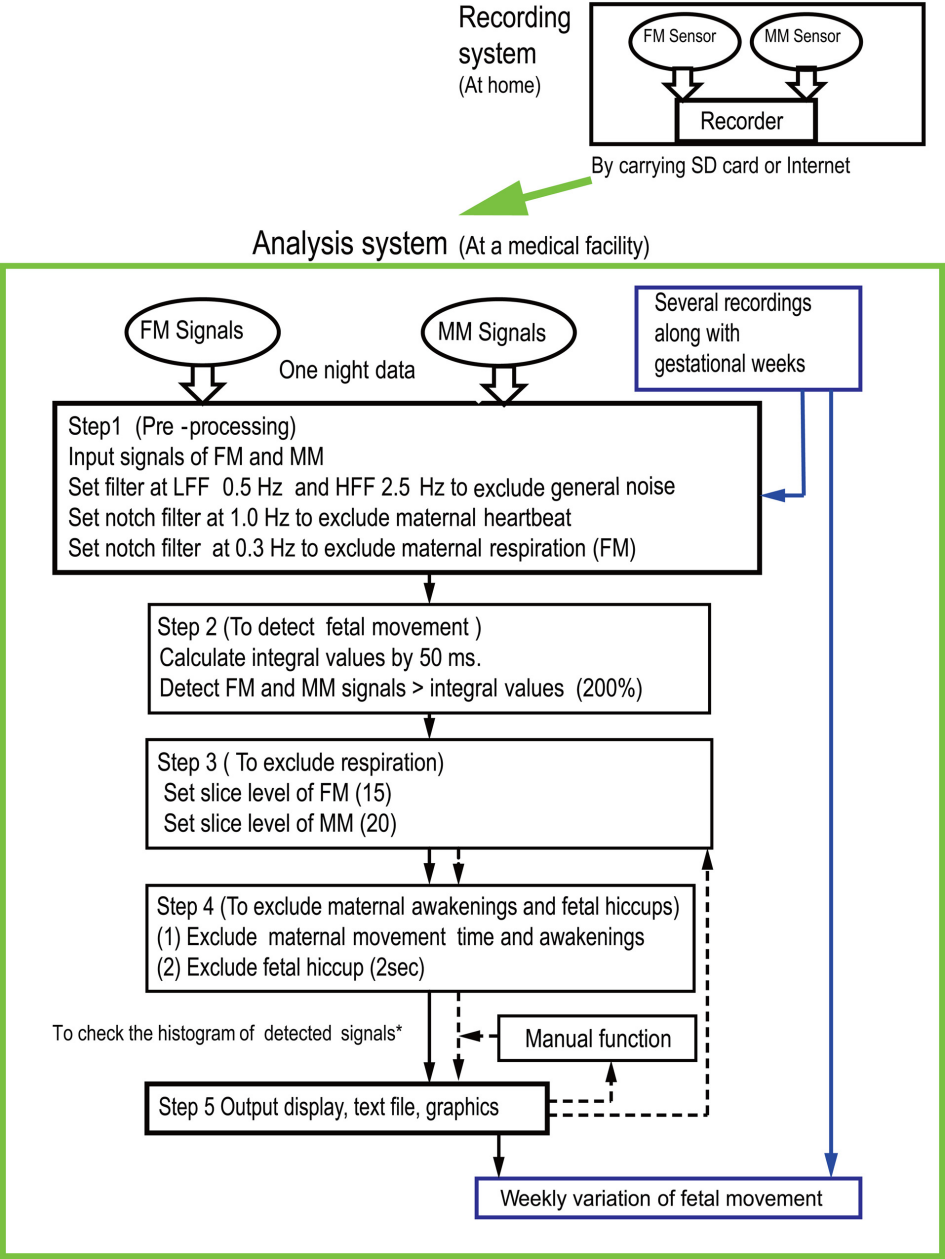
The FMAM recording-analysis system and a flow chart. The pregnant woman records fetal movement during her sleep at home. She can bring the SD data card to her medical facility or send it by the Internet. The analysis system itself is at the medical facility. The flow chart of the analysis system is explained in Section 3 of Methods. The mark (*) is also explained in Step 3 of Section 3. Broken lines are used for data that include many artifacts.

The FMAM sensor and recorder were described in detail in our previous studies [10, 11]. We will introduce them only briefly here (Fig 1). The recorder consists of two channels with two sensors: one to record fetal movement (FM channel), and the other to record maternal movement (MM channel). The sensors pick up capacitive acceleration changes from maternal and fetal movements. That means the sensors pick up variation fetus and mothers moved. The fetal movement sensor (FM sensor) has high output power, 720mV/m/s^2^, while the maternal movement sensor (MM sensor) has one-fifth the FM sensor's sensitivity (120mv/m/s^2^). The MM sensor is used to exclude maternal movement from the FM sensor channel. The recorder [11] uses Windows XP or Windows 7. The unit weighs 290 g.

The FMAM recorder can be operated by the woman herself. She takes it home to record fetal movement as she sleeps. Just before going to bed, she uses adhesive and surgical tapes to attach the two sensors: one on her abdomen where she most strongly feels fetal movement (FM sensor), and the other on her thigh (MM sensor). She turns on the recorder and goes to bed; the next morning, she turns it off.

### 2 The basic features of the software

**Fig 2:**
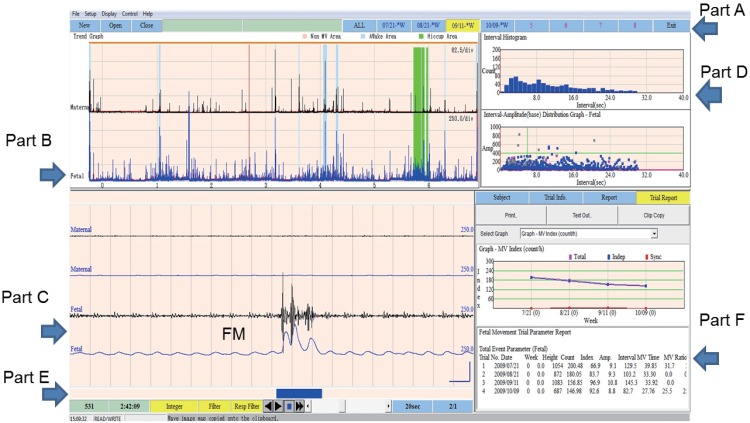
Screen shot. This screen shot shows the basic feature of the software. **Part A**: A display of all gestational weeks can be seen with one click. **Part B:** These lines show modified movement signals (integral values) for the mother (above, in black) and the fetus (below, in blue) for one night. Light blue shows maternal wake time, and green shows fetal hiccup movements. **Part C:** These lines show the signal from the accelerometer (above) and integral (below) for the mother (top two lines) and the fetus (bottom two lines). This is for a 20-second period. The heavy blue line at the bottom indicates a significant moment. FM represents fetal movement. **Part F:** We can call up the results from any week. We can calculate the fetal parameters (for example, the number of fetal movements per night or per hour).

To analyze the recorded fetal movement signals, we wrote an original software program using Windows Visual C. We did not use any other commercial tools. We show the screen shot for data of a 29-year-old woman at 32 gestational weeks (Fig 2). The following six features for data analysis can be seen:

1. The ability to analyze multiple nights of recorded data for the same woman in order to see week-by-week changes (Part A)
2. The ability to see a condensed display of one night's data at a glance (Part B)
3. The ability to display the raw data (Part C)
4. The ability to check a histogram of the detected signal distributions to see whether artifacts were included (Part D)
5. The ability to make manual corrections (Part E)
6. The ability to export the results to other programs such as Excel or Word (Part F)

### 3 The process of analysis and the rejection of artifacts

It is important that we not only detect real fetal movement signals but also remove artifacts, such as the mother's heartbeat, respiration, and body movement. Fig 1 shows the process of analysis from pre-processing the input signal to excluding maternal physiological artifacts. The steps are as follows:

#### Step 1 (pre-processing)

There are two channels, one for the fetal signal and one for the maternal signal. The signal from each channel is filtered through a low frequency filter (LFF) of 0.5Hz (-6dB/oct) and a high frequency filter (HFF) of 2.5Hz (-12 dB/oct) to exclude general noise. We set a notch filter in the MM and FM channels at 1.0 Hz to exclude the maternal heartbeat, and in the FM channel, we set a notch filter at 0.3 Hz to exclude maternal respiration.

#### Step 2 (detecting fetal movement)

**Fig 3:**
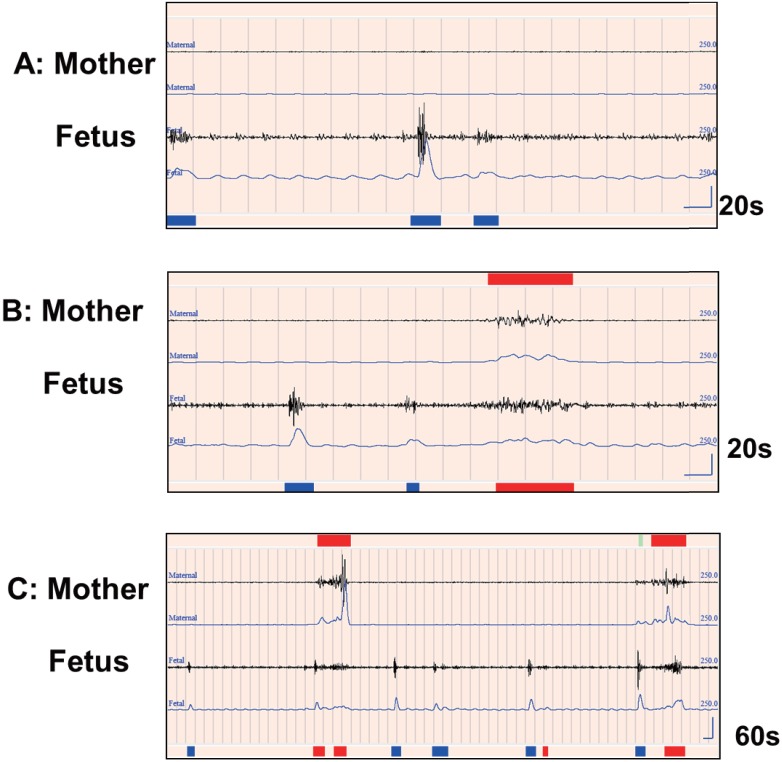
Fetal movement signals and maternal artifacts. The heavy blue lines at the bottom indicate fetal movement identified by the automated analysis software. The heavy red lines indicate rejections. The two upper lines show maternal movement. The two lower lines show fetal movement. For each pair, the upper line is from the accelerometer after passing the filters, and the lower line is the modification (integral). One grid mark indicates one second with 20 or 60 seconds per screen. **A:** This shows fetal movement and the influence of the maternal heartbeat in the FM channel (29 yrs, 32 gestational weeks). Since there is no movement by the mother, the signals on the FM channel are recorded as fetal movement. **B:** This shows maternal movement influencing the fetal movement channel. In this case (29 yrs, 32 gestational weeks), the signals on the FM channel include both maternal and fetal movements, so the maternal movement is excluded by the software. **C:** This shows periodic leg movements by a mother (26 yrs, 24 gestational weeks) and their influence on the fetal signal. Here the mother (upper two lines) has periodic leg movement at intervals of 20 to 40 seconds. The screen shows 60 seconds. Two (thigh) movements are seen in the top line for the mother. They influence the abdomen through vibration, reflected in the modified signal (integral) shown in the second line. In cases such as this, the mother's body movement signals can be removed by the analysis software.

The input signals are then changed to integral values per 50 ms. If the signals’ integral amplitude values are higher than twice the average amplitude value during the three seconds just before and after (we say 200%), a fetal movement signal is detected. Judging from a number of experimental trials, this method successfully excludes maternal heartbeat from the FM channel (Fig 3A).

#### Step 3 (excluding maternal respiration)

**Fig 4:**
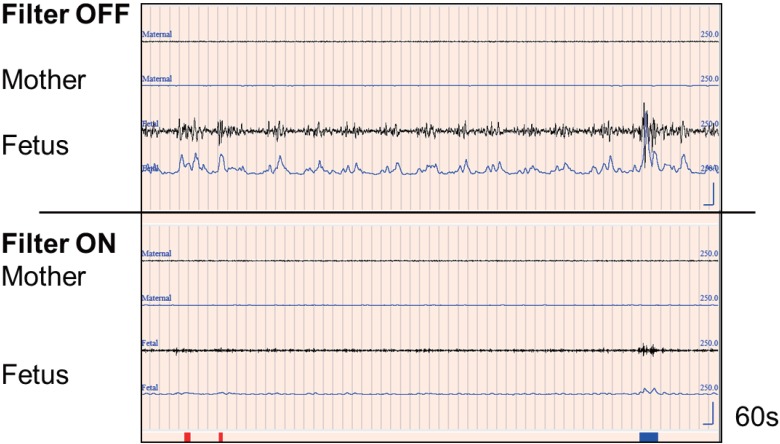
Maternal respiration movement. This figure shows maternal respiration influencing the FM channel (37 yrs, 36 gestational weeks). One grid mark indicates one second with 60 seconds per screen. The upper window shows the influence of the mother's breathing (3–5 sec intervals). We made two functions in order to remove respiration artifacts and explained in Step 3. The final decision is shown by the heavy blue line; the red lines indicate removal by the minimal threshold level.

Fig 4 shows artifacts from maternal breathing, which can easily be mistaken for fetal movement. Two steps are necessary to reject maternal breathing artifacts. First we set a notch filter at 0.3 Hz; then, we set a minimum threshold level. During Rapid Eye Movement (REM) sleep, there are large changes in the autonomic nervous system, which includes heart rate and respiration. Maternal breathing movements occur at 3–5 sec intervals and appear as artifacts in the FM channel during maternal REM sleep. Thus, we could not exclude them with only a 0.3 Hz notch filter. We also had to set a minimum threshold level. We tried various threshold levels for the integral values and then referred to the distribution of the intervals between detected signals using the histogram tool (in Part D in Fig 2). The histogram shows the frequency of the various intervals between detected signals. It ranges from 0 sec to 40 sec with steps every 1.0 sec. When the frequency counts for the 3.0 sec, 4.0 sec, and 5.0 sec columns were extremely high, the detected signals included respiration. In that case, we had to change the level of the threshold integral value. After many trials, we found that the optimal values for obtaining fetal movement data with minimal artifacts were 15 for the FM channel and 20 for the MM channel.

#### Step 4 (maternal awakening, leg movement, and fetal hiccups)

We reject the signal in the FM channel when maternal movement signals are detected in the MM channel (Fig 3B). When maternal awakening is brief (less than four times in one minute), it is not removed from the analysis time. Restroom time, however, is longer, and we excluded the entire time from the total used in the data analysis.

Manconi et al. reported that 26 percent of pregnant women have restless leg syndrome [13]. Periodic limb movement during sleep (PLM) is often seen in pregnant women with restless leg syndrome. According to the criteria of the American Sleep Disorder Association, periodic leg movement is defined as repetitive muscle jerks lasting from 0.5 to 5 seconds, repeated at intervals ranging from 5 to 90 seconds [14]. We set duration of 1.0 sec in the MM signal to detect maternal periodic limb movements. PLM also influences the FM channel, so signals arising from PLM can be rejected (Fig 3C).

**Fig 5:**
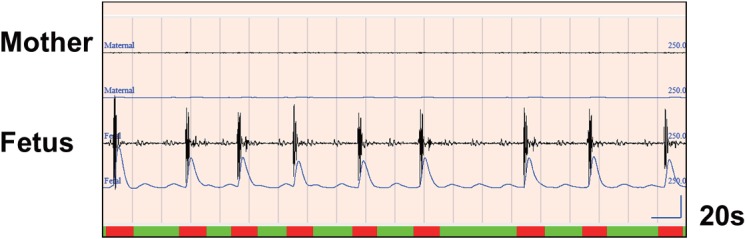
Fetal hiccup movements. This figure shows fetal hiccup movements (29 yrs, 32 gestational weeks). Fetal hiccup movements are identified by the software and are not counted as fetal movement. The two bottom lines show the fetal hiccup movements, and the areas marked with heavy red lines are excluded. The heavy green line shows the overall period of hiccup movement, which generally ranges from 5 to 15 minutes.

Fetal hiccups have a characteristic movement with an interval of about two seconds and often continue for 5–15 minutes (Fig 5). Fetal hiccup movement is physiologically different from gross fetal movement. If we include fetal hiccups in the analysis, they are about 30 percent of the number of fetal movements for one night, and the results confuse the meaning of gross fetal movement. To detect fetal hiccups, we set the duration of the FM signal at 0.2 sec. After detecting such a hiccup signal, the software excludes occasions with more than 15 signals per minute.

#### Step 5 (output display, text file, and graphics)

We obtain fetal movement data through these four steps. It is then necessary to check the histograms of the detected signals’ intervals. If the 3–5 sec columns and the 2 sec column in the histograms of detected signals’ intervals are extremely high, it means the detected signals include signals of respiration and hiccups. We can confirm this by looking at the raw signals without the filter set at 0.3Hz. We then analyze the data by changing the slice level for fetal movement amplitude in Step 3 or by using the manual function.

We obtain real fetal movement signals through these five steps because we can remove the maternal signal, leaving only the fetal. This gives us the number of fetal movements per hour and per night.

A woman can record fetal movement several times during gestation, for example at 24, 28, 32, and 36 weeks, and the software can display the results of several recordings. Weekly variation in fetal movement contributes to an evaluation of fetal well-being.

### 4 Experiment I

We used the data for six healthy pregnant women (26–36 yr) at 28–38 weeks as previously reported by Ryo and Kamata [12]. The subjects had no complications in pregnancy and delivery. Their Body Mass Index (BMI) ranged from 17.5 to 22.9. All the newborns were delivered at term without anomalies or neurological problems (weight: 2740–3438 g). The subjects did 61 recordings of fetal movement overnight according to their own irregular schedules. The recording method is described in Section 1 of Methods above. Manual analysis results of weekly variation in fetal movement were reported [12]. Fetal movements were judged to be present during 10-sec epochs when their amplitude exceeded the maternal heartbeat [12], the same method as in our previous study comparing manual analysis with ultrasonography [11]. The maternal heartbeat has the largest amplitude of all maternal signals except maternal movement.

The data were first analyzed using the new automated software with the default values described above. Next, we compared the results detected automatically with the results detected manually. Agreement between the manual and automated results for the same 10-sec epochs for one subject during one night was calculated and then evaluated by prevalence-adjusted bias-adjusted kappa (PABAK) [15]. PABAK takes into consideration quantitative indicators of the differences in rate of occurrence of fetal movement and on the analysis methods. To describe the relative strength of agreement associated with kappa statistics, the following labels are usually assigned to the corresponding ranges of kappa: 0.00, poor; 0.00–0.20, slight; 0.21–0.40, fair; 0.41–0.60, moderate; 0.61–0.80, substantial; 0.81–1.00, almost perfect [16].

#### Ethics statement

The study was approved by the ethics committee at Teikyo University, School of Medicine (Number: 13–100). All the subjects gave written informed consent to the study.

### 5 Experiment II

The subjects were twelve healthy pregnant women (29–39 yr) at 24–36 gestational weeks. The subjects had no complications in pregnancy and delivery. Their BMIs ranged from 17.5 to 22.1. All newborns were delivered at term without anomalies or neurological problems (weight: 2566–3444 g). We asked the women to record fetal movement on a fixed schedule, once every four weeks, from 24 to 36 weeks of gestation. On four occasions for three subjects, there were no recordings, due to their schedules, so we had a total of 44 recording sessions. The mothers were taught how to record fetal movement, as described above.

We used the default values in Experiment I and analyzed the data with the automated software. We examined fetal movement week by week following gestation to establish a new fetal movement count index. We calculated the number of fetal movements per hour and compared over the four sessions (at 24, 28, 32 and 36 weeks) using a one-way analysis of variance with repeated measures [17]. Scheffé tests were used for a post-hoc test [17].

#### Ethics statement

Three ethics committees (Tokyo Institute of Psychiatry: Tokyo Metropolitan Institute of Medical Science, Number 22–16; St. Luke’s College of Nursing, Number 08–064; and the Institute for Science of Labour, Number 11–002) approved this experiment. All the mothers gave written informed consent to the study.

## Results

### 1 Real fetal movement detection and artifact removal

Fig 3A shows real fetal movement signals. The mother is not moving, so these show only the movement of the fetus. Fig 3A includes maternal heartbeat signals in the FM channel. Setting the integral value at the 200% level lets us reject the heartbeat signals. The heartbeat integral value is clearly regular, while the fetal movement signal is more like bursts.

Fig 3B shows fetal movement and maternal body movement. When there are maternal movement signals detected in the MM channel, simultaneous signals detected in the FM channel are rejected. PLM also appears in the FM channel, so those signals are also excluded (Fig 3C).

Fig 4 shows signals from maternal breathing. The FM sensor picks up the mother’s irregular abdominal breathing, which is often seen during REM sleep. This is characteristic of late pregnancy. We made two adjustments to remove these respiration artifacts. One is setting the notch filter at 0.3 Hz; the second is setting a minimum threshold level. The notch filter is effective (Fig 4). We tried various threshold levels for the integral, using the histogram tool, and found that an initial integral value of 15 is optimal for minimizing breathing artifacts.

Fig 5 shows fetal hiccups, which are movements continuing for 5–15 min at 2-sec intervals. Our definition of more than 15 movements per minute could exclude typical hiccups. However, fewer than 15 hiccups per minute were not excluded. This usually occurs at the beginning and ending of a hiccup session.

### 2 Experiment I

**Fig 6:**
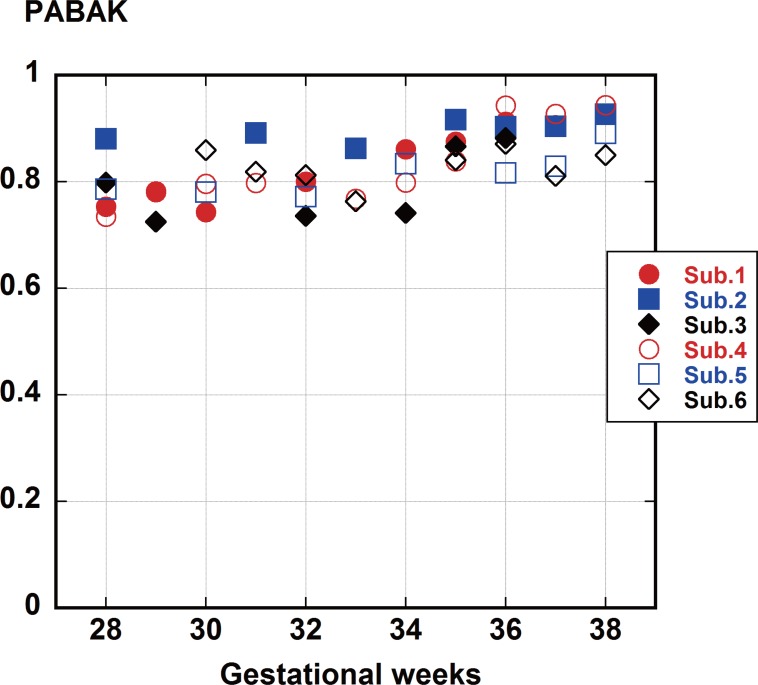
Agreement between manual and automated detection for the six subjects. The mean and SD of the agreement for the six subjects were 0.83 and 0.04, which can be interpreted as almost perfect.

One PABAK value for each subject was calculated for each night. The mean (SD) values for each of the six subjects’ PABAK were 0.83 (0.06), 0.90 (0.02), 0.79 (0.06), 0.85 (0.08), 0.81 (0.04), and 0.83 (0.03). The mean (SD) PABAK value for all six subjects was 0.83 (0.04). Each subject’s PABAK values tended to increase in late pregnancy (Fig 6).

### 3 Experiment II

**Fig 7:**
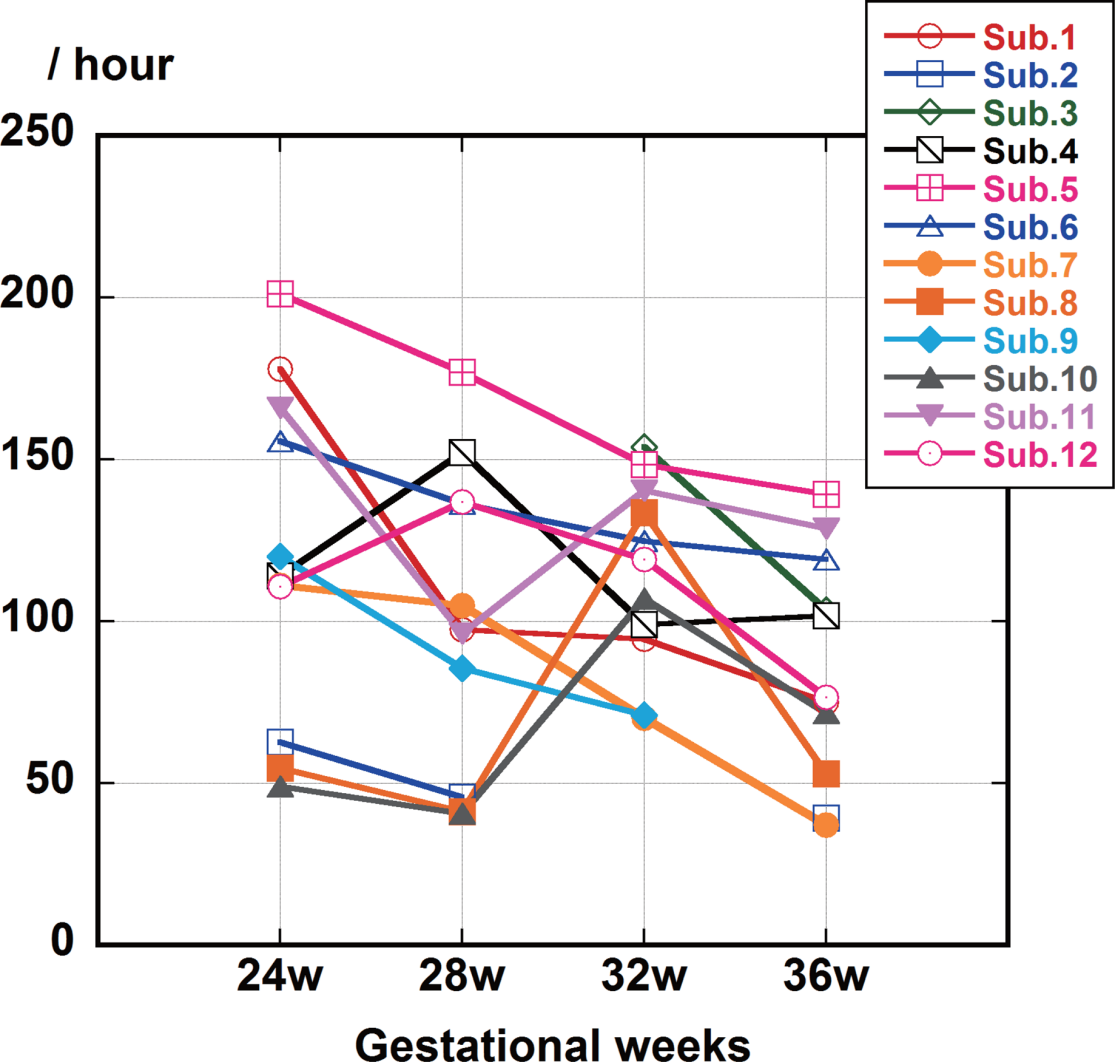
Fetal movements per hour from week to week during gestation. Mean values for the number of fetal movements per hour at night decreased from 24 weeks to 36 weeks (p<0.02).There were significant differences in fetal movement counts per hour from week to week with large individual differences (p<0.001).

Fig 7 shows the count values for fetal movement per hour during each gestational week. Mean and SD count values for fetal movement detected per hour were 120 (50) at 24 weeks, 101 (51) at 28 weeks, 115 (46) at 32 weeks, and 86 (29) at 36 weeks. There were significant differences (F (3, 33) = 3.68, p<0.02) in count values for fetal movement per hour for each of the four sessions. There were also large individual differences (F (11, 33) = 5.12, p< 0.001). There was a significant difference in count values for fetal movement per hour between 24 weeks and 36 weeks using post-hoc Scheffé tests (p<0.04).

## Discussion

### 1 Accurately detecting fetal movement

In our previous study in 2012, we confirmed high agreement between fetal movement detected by ultrasound and that detected by the FMAM device [11]. In late pregnancy, 30–39 weeks, the mean and SD for the PABAK were 0.83 and 0.06, which is almost perfect.

However, these earlier data were recorded by subjects who were at rest and kept quiet in a semi-Fowler position, so there were minimal artifacts. In addition, the recording time was short, about 30 minutes. The present study was conducted under normal life conditions in which the pregnant women slept freely at night. Long-term home monitoring includes many artifacts, such as maternal movement to change sleeping position or to get up to go to the restroom, as well as heartbeat and breathing changes during REM sleep. The FMAM device software successfully minimized maternal physiological artifacts.

Since the FMAM device has an MM channel, maternal movement overlapping the FM channel can be excluded, even for pregnant women with periodic limb movements. Maternal heartbeat is also excluded by a process of comparing integral values. However, it is more difficult to remove maternal breathing movement. After many trials, we set the notch filter at 0.3 Hz and set a minimal threshold value for rejecting breathing movement. These two steps were effective in excluding breathing movement artifacts. When the minimum threshold integral value is set at 15, most breathing artifacts can be rejected. When we set it higher than 15, the software began removing real fetal movement. On the other hand, when we did not set any minimum threshold value, there was an extremely high peak at 3–5 seconds in the histogram of detected signals. That meant maternal breathing movements were not being excluded.

Fetal hiccup movement is defined as more than 15 movements per minute, and fetal hiccups cannot be completely excluded. When the histogram of the signals shows a high count level at 2 sec, we must exclude the hiccups manually.

### 2 Experiments I and II

In Experiment I, the mean (SD) value for PABAK between manual and automated analyses was 0.83 and 0.04, which we interpreted to be almost perfect. The results of a manual analysis of the data were previously reported to be similar to an analysis done by ultrasonography [11]. Therefore, the high PABAK value means that the results of the automated analysis, using the initial default values, correspond well to the results reported by ultrasonography. The 10-sec epoch analysis of fetal movement used in the manual analysis contained the authors’ differences of opinion, even though the definition of fetal movement was clear. Another difference was that the manual analysis included non-movement time. We are now documenting normal variation in order to develop a new index for the number of fetal movements per hour during maternal sleep throughout pregnancy.

In Experiment II, significant variation in fetal movement by gestational week was observed. A previous manual study [12] showed that the percent of fetal movement during maternal night sleep decreased from 30–34 weeks to 35–38 weeks, and in the present study, mean values for the number of fetal movements per hour at night decreased from 24 weeks to 36 weeks (p<0.02). The weekly decrease reported by our automated software corresponded well with the previous manual study. Significant individual differences were also observed (p<0.001), including developmental differences. The number of fetal movements at 24–32 gestational weeks was about 100 per hour, and that value is reasonable based on our short-term ultrasound study [11]. In that study, we observed 107.8 fetal movements per hour. [11].

Blyton et al. recently reported that the number of fetal movements per hour at night was 100 during Non Rapid Eye Movement (NREM) sleep and that it increased to 150 per hour during REM sleep for a control group of pregnant women [18]. There were differences between Blyton’s methods and ours, but our count matched their count during NREM sleep on the control subjects at 33 gestational weeks. We would like to point out that in Blyton’s study, the higher fetal movement number of 150 during REM sleep might be due to maternal breathing artifacts. The breathing rate during REM sleep is irregular, and our FM sensor picks up those changes in acceleration. In our previous study, we simultaneously recorded fetal movement and polysomnograms, including electroencephalograms (EEGs) and respiration [10]. At that time, we found that our sensor picked up a great deal of maternal respiration during REM sleep. In our present study, there were many breathing artifacts during the second half of nocturnal sleep. REM during sleep occurs more often in the second half of night sleep than in the first half [19]. We must be aware of respiration artifacts occurring during the second half of nocturnal sleep.

In Experiment II, we show that there are weekly variations and significant individual differences in fetal movement per hour. The peak count of fetal movement was different for each fetus (Fig 7). These findings mean there are large differences in fetal development from 24 to 36 gestational weeks. Fetal development must be considered when observing fetal movement.

All the data in Experiments I and II were obtained from healthy women with BMIs between 17.5 and 22.9. The validity of the FMAM system is limited to healthy pregnant women with normal range BMIs. We must pay attention to each pregnant woman’s individual situation, including obesity and an anterior placenta. Obese pregnant women tend to have sleep apnea, which can cause an increase in respiration artifacts. Experiment I included women with anterior placentas, and agreement between manual and automatic analyses was obtained; however, we could not demonstrate the specific effects of an anterior placenta. The amplitude of fetal movement signals may be reduced. In future studies, we must clearly demonstrate any influence on the FMAM system caused by individual differences among pregnant women.

### 3 Home monitoring of fetal movement

The pregnant women in our study were able to easily record fetal movement by themselves at home at night using the FMAM recorder, and we were able to effectively use the new analysis system. Using this system to record and analyze fetal movement along gestational weeks will be useful in clinical field work in order to monitor fetal well-being.

Ryo et al. reported the case of a pregnant woman who underwent an emergency caesarean delivery at 31 gestational weeks because of a non-reassuring fetal heart rate pattern. The woman delivered a female neonate weighing 1312 g. She had recorded fetal movement by herself during sleep using the FMAM at 30 gestational weeks, and the result of a manual analysis showed decreased fetal movement as compared with the control data reported previously [20]. Though the case suggests that the FMAM system may be useful clinically, it will be necessary to collect a great deal of control data on healthy pregnant subjects. We need to know the normal variation in movement during fetal development.

A recent study of questionnaires on maternal sleep practices reported that the risk of stillbirth among pregnant women sleeping on the left side was lower than the risk among women sleeping supine or on the right side [21]. The reason was thought to be related to the anatomical position of the inferior vena cava and the aorta. However, as this was only a questionnaire study, this hypothesis should be reexamined. If home monitoring for pregnant women becomes more widely available, such a reexamination would be possible.

The FMAM recorder also seems to empower women. While conducting these experiments, we noticed a positive effect on the women themselves due to their active involvement in the monitoring process. They said that they were paying much more attention to fetal movement than they were before joining in the experiments. The FMAM system not only provides an objective measure of fetal activity, but also improves the mothers’ awareness and concern. It has not been clearly demonstrated that fetal movement counting by pregnant women decreases the number of stillbirths; however, we know that maternal perception of reduced fetal movement is one important sign of fetal compromise [22]. Thus, we believe that the FMAM system will become a good screening tool that provides reliable home information to a medical facility.

In the future, if home monitoring with the FMAM recorder and its automated analysis system can be established, fetal development during gestation can be observed over the long term, and this will open new fields of research in such areas as neurophysiological studies on the relationships between cerebral palsy and hypoxic ischemic encephalopathy, and neurophysiological studies on fetal development.

## Conclusion

The agreement between manual and automated signal analysis is high, and the new software can be used to accurately count fetal movements and successfully minimize the number of artifacts. This method of detecting and analyzing fetal movement is appropriate for monitoring fetal well-being from week to week during gestation.

## References

[1] Sadovsky E, Yaffe H. Daily fetal movement recording and fetal prognosis. Obstet Gynecol. 1973;41: 845–850. 4196643

[2] Pearson JF, Weaver JB. Fetal activity and fetal wellbeing: an evaluation. BMJ. 1976; 29: 1305–1307. 10.1136/bmj.1.6021.1305 PMC1640347 1268677

[3] Manning FA, Plat LD, Sipos L. Antepartum fetal evaluation: development of a fetal biophysical profile. Am J Obstet Gynecol. 1980;136: 787–795. 7355965 10.1016/0002-9378(80)90457-3

[4] Maeda K, Tatsumura M, Utsu M. Analysis of fetal movements by Doppler actocardiogram and fetal B-mode imaging. Clinics in Perinatology. 1999;26: 829–851. 10572724

[5] Besinger RE, Johnson TRB. Doppler recordings of fetal movement: Clinical correlation with real-time ultrasound. Obstet Gynecol. 1989;74: 277–280. 2664613

[6] Goldenberg RL, McClure EM, Bhutta ZA, Belizan JM, Rubens CE, Mabeya H, Flenady V, Darmstadt GL. Lancet’s Still births Series steering committee. Stillbirths: the vision for 2020. Lancet. 2011;377:1798–1805. 10.1016/S0140-6736(10)62235-0 21496912

[7] Morokuma S, Fukushima K, Otera Y, Yumoto Y, Tsukimori K, Ochiai M, Hara T, Wake N. Ultrasound evaluation of fetal brain dysfunction based on behavioral patterns. Brain Dev. 2013;35: 61–67. 10.1016/j.braindev.2012.01.007 22321861

[8] DiPietro JA, Kivlighan KT, Costigan KA, Rubin SE, Shiffler DE, Henderson JL, Pillion JP. Prenatal antecedents of newborn neurological maturation. Child Dev. 2010;81: 115–130. 10.1111/j.1467-8624.2009.01384.x 20331657 PMC2846092

[9] Koyanagi T, Horimoto N, Takashima T, Satoh S, Maeda H, Nakano H. Ontogenesis of ultradian rhythm in the human fetus, observed through the alternation of eye movement and no eye movement periods. J Reprod Infant Psycho. 1993;11: 129–134.

[10] Nishihara K, Horiuchi S, Eto H, Honda M. A long-term monitoring of fetal movement at home using a newly developed sensor: An introduction of maternal micro-arousals evoked by fetal movement during maternal sleep. Early Hum Dev. 2008;84: 595–603. 10.1016/j.earlhumdev.2008.03.001 18450390

[11] Ryo E, Nishihara K, Matsumoto S, Kamata H. A new method for long-term home monitoring of fetal movement by pregnant women themselves. Med Eng Phys. 2012;34: 566–572. 10.1016/j.medengphy.2011.09.001 21962570

[12] Ryo E, Kamata H. Fetal movement counting at home with a fetal movement acceleration measurement recorder: A preliminary report. J Matern Fetal Neonatal Med. 2012;25: 2629–2632. 10.3109/14767058.2012.704449 22734497

[13] Manconi M, Govoni V, De Vito A, Economou NT, Cesnik E, Casetta I, Mollica G, Ferini-Strambi L, Granieri E. Restless legs syndrome and pregnancy. Neurology. 2004;63: 1065–1069. 15452299 10.1212/01.wnl.0000138427.83574.a6

[14] American Sleep Disorder Association. Recording and scoring leg movements. The atlas task force. SLEEP. 1993;16: 748–759. 8165390

[15] Byrt T, Bishop J, Carlin JB. Bias, prevalence and kappa. J Clin Epidemiol. 1993;46: 423–429. 8501467 10.1016/0895-4356(93)90018-v

[16] Landis JR, Koch GG. The measurement of observer agreement for categorical data. Biometrics. 1977;33: 159–174. 843571

[17] Winer BJ. (1971) Statistical principles in experiment design McGraw-Hill, New York pp 261–308.

[18] Blyton DM, Skillton MR, Edwards N, Hennessy A, Celemajer DS, Sullivan CE. Treatment of sleep disordered breathing reverses low fetal activity levels in preeclampsia. SLEEP. 2013;36: 15–21C. 10.5665/sleep.2292 23288967 PMC3524539

[19] Williams RL, Harman W, Agnew W, Webb WB. Sleep patterns in young adults: An EEG study. Electroenceph clin Neurophysiol.1964;17: 376–381. 14236819 10.1016/0013-4694(64)90160-9

[20] Ryo E, Kamata H, Seto M. Decreased fetal movements at home were recorded by a newly developed fetal movement recorder in a case of a non-reassuring fetal status. J Matern Fetal Neonatal Med. 2014;27: 1604–1606. 10.3109/14767058.2013.863868 24195670

[21] Stacey T, Tompsom JMD, Mitchell EdA, Ekeroma AJ, Zuccollo JM, McCowan LME. Association between maternal sleep practices and risk of late stillbirth: a case control study BMJ. 2011;14: 342–347. 10.1136/bmj.d3403 PMC3114953 21673002

[22] FrØen JF. A kick from within-fetal movement counting and cancelled the progress antenatal care. J Perinat Med.2004;32: 13–24. 15008381 10.1515/JPM.2004.003

